# Handbooks for Senior Students and Practitioners

**Published:** 1908-09-05

**Authors:** 


### HANDBOOKS FOR SENIOR STUDENTS AND PRACTITIONERS.

### MEDICINE.

"Allbutt's System of Medicine." 8 vols., 25s. each, Macmillan and Co. (A new edition, edited by Professor Clifford Allbutt and Dr. H. D. Rolleston is in course of publication, and several volumes have already appeared. "Allbutt" is deservedly ranked as one of the finest works on medicine, and is especially valuable for practitioners because of the great amount of space given to consideration of treatment and pathology.)

"Osler's System of Medicine." Edited by Osler and McCrae. (7 vols.). 24s. each volume. Oxford Medical Publications, 20 Warwick Square, E.C. (The series is not yet complete, but, judging from those that have already appeared, the system is likely to prove one of the best which it is possible to obtain.)

"Allchin's Manual of Medicine." 5 vols., vols. i. to iv. 7s. 6d., vol. v. 10s. Macmillan and Co. (Shorter but very useful, and particularly good on diseases of special systems.)

"Osler's Practice of Medicine." 1 vol. Appleton. 21s. net. (An admirable text-book of medicine; well written and comprehensive.)

"Roberts' Medicine." 26s. 2 vols. H. K. Lewis, Gower Street. (Fully up to date and practical; well written.)

"Taylor's Medicine." Churchill, 7 Great Marlborough Street, 15s. net. (A useful manual; exceedingly popular with students.)

"Savill's Medicine." 2 vols., 12s. 6d. each. Churchill.

"Fagge and Pye Smith's Medicine." 2 vols. 42s. net. Churchill. (For many years a standard text-book.)

"Whitla's Manual of Medicine." 2 vols. £1 12s. Henry Renshaw. (Sir Wm. Whitla's new work, in dictionary form; concise and practical.)

"Carter's Elements of Practical Medicine." 1 vol. 10s. 6d. H. K. Lewis. (A popular condensation, probably the best of its kind.)

"Bury's Clinical Medicine." 1 vol. 16s. Charles Griffin and Co., Exeter Street, London.

"Monro's Medicine." Baillière, Tindall, and Cox. 1 vol. 15s.

### SURGERY.

Walsham's and Spencer's "Theory and Practice of Surgery," 1 vol. 18s. net. Churchill. (Contains a very large amount of information in comparatively few pages, and for this reason it is a little difficult to read in at all a *dilettante* fashion.)

Rose and Carless' "Manual of Surgery," 1 vol. 21s. net. Baillière, Tndall, and Cox. (This text-book runs neck and neck with the preceding in popular favour. Its *format* is perhaps a little more pleasing than that of its rival.)

Cheyne and Burghard's "Manual of Surgical Treatment," in six parts, 9s. to 18s. each vol. Longmans, Green. (Very complete, thoroughly practical, and in every way satisfactory for a senior student.)

Jacobson and Steward's "Operations of Surgery," in two volumes. Churchill. 42s. net. (Perhaps the most satisfactory English text-book of operative surgery extant. The fifth edition has now appeared, edited by R. P. Rowlands.)

"A System of Practical Surgery," edited by Profs. Von Bergmann, Bruns and Mikulicz, translated into English and edited by Drs. Bull, Frost, Flint, and Martin, in five vols. £6 6s. net. Williams and Norgate. (This system gives a very complete account of the science and art of surgery at the present time.)

"A System of Surgery," edited by Sir Frederick Treves, in two volumes, 48s. Cassell and Co. (The work consists of an excellent series of articles by surgeons attached to the various London hospitals, without any attempt to make it encyclopædic. A new edition would be appreciated. It may be supplemented by the new edition of Treves' "Manual of Operative Surgery," revised by the author and Mr. J. Hutchinson, jun., which is also published in two volumes by Messrs. Cassell, price 42s.)

---

*Surgical Anatomy and Surgical Pathology* form the backbone of surgery as a science. But too many students are content to learn only so much as is sufficient to enable them to obtain a minimum examination knowledge. There are several good English text-books on surgical anatomy. One of the best is M'Lachlan's "Applied Anatomy: Surgical, Medical, and Operative," in two volumes, published at Edinburgh, by Messrs. E. and S. Livingstone. It is clear, well written and well illustrated.

Rawling's "Landmarks and Surface Markings of the Human Body," published by H. K. Lewis, 5s. net, is somewhat on the lines of the late Mr. Holden's "Medical and Surgical Landmarks." (Mr. Rawling's book is well illustrated, and has already reached a second edition.)

Sir Frederick Treves' "Surgical Applied Anatomy" has long been a favourite with students. A revised edition has recently been issued by Dr. Arthur Keith, and published by Messrs. Cassell and Co. It is in one volume, well illustrated.

Box and McAdam Eccles' "Clinical Applied Anatomy" (Churchill, 12s. 6d. net), is in many respects an interesting and useful volume, reminding the reader a little of Hilton's "Rest and Pain," which is so well known to every thoughtful student of surgery.

Bowlby's "Surgical Pathology" (Churchill, 10s. 6d. net) contains a large number of drawings, and describes in simple language the various pathological processes with which the student of surgery should be acquainted. It is a useful and popular handbook.

### OBSTETRICS AND GYNÆCOLOGY.

Galabin's "Manual of Midwifery" (Churchill, 14s.). A thoroughly popular and useful handbook, practical and concise. Dakin's "Handbook of Midwifery" (18s.) is another favourite with students and practitioners. Eden's "Manual of Midwifery" (Churchill, 10s. 6d. net) is perhaps the best small book for students.

Whitridge Williams's "Text-book of Obstetrics." An excellent work, well illustrated, by an American obstetrician. Deservedly popular in this country.

Herman's "Difficult Labour" (Cassell, 12s. 6d.). An indispensable work.

Herman's "Diseases of Women" (Cassell, 1 vol., 25s.).

A standard work on gynæcology. It approaches the subject from the clinical and practical standpoint, and is stamped throughout with the author's personality. Lewers' "Practical Text-book of the Diseases of Women" (Lewis, 10s. 6d.) is a smaller work, popular with students, but less suited to the practitioner. Galabin's "Diseases of Women" (Churchill, 16s. net) is a well-written, useful text-book of gynæcology for students and practitioners.

### PATHOLOGY, HYGIENE, &c.

"Green's Pathology" (1 vol., Renshaw, 18s. net, 10th edition, by W. C. Bosanquet). A reliable introduction to pathology, well illustrated, readable, and up-to-date. "Martin's Pathology" (15s. net) is also an excellent manual; while Bland-Sutton's "Tumours" (Cassell, 21s.) is an interesting book which all students should read.

Muir and Ritchie's "Manual of Bacteriology" (Henry Frowde and Hodder and Stoughton, 12s. 6d.). Perhaps the most useful work of its kind. A good introduction to the science of bacteriology.

Emery's "Clinical Bacteriology and Hæmatology" (Lewis, 7s. 6d.) is specially written for practitioners.

Hutchison and Rainey's "Clinical Methods" (Cassell and Co., 10s. 6d.). Almost indispensable to the clinical clerk and the scientific physician. In connection with the subject of clinical methods Dr. Gee's "Auscultation and Percussion" (Oxford Medical Publications, 5s. net) should be mentioned. It is a book which every senior student should procure and keep with him throughout his professional career.

Among useful works on Hygiene and Public Health are Whitelegge and Newman's "Hygiene and Public Health" (Cassell, 7s. 6d.), Hamer's "Manual of Hygiene" (Churchill, 12s. 6d.), Parkes and Kenwood's "Hygiene and Public Health" (12s.), and Ham's "Handbook of Sanitary Law" (2s. 6d.).

The most generally useful and popular handbooks on Forensic Medicine are Dixon Mann's "Forensic Medicine and Toxicology" (21s.), Husband's "Forensic Medicine, Toxicology, and Public Health" (edited by Buchanan and Hope, 10s. 6d. net), and Taylor's large "Principles and Practice of Medical Jurisprudence" (revised by F. J. Smith, 36s. net).

### SPECIAL BRANCHES OF MEDICINE AND SURGERY.

*Diseases of Children.*—Among medical works those of Goodhart and Still (Churchill, 12s. 6d.), Eustace Smith (Churchill, 22s.), and Cautley (Churchill, 7s. 6d.) should be mentioned.

Ashby and Wright's "Diseases of Children" (Longmans, Green, 21s. net) is convenient because it contains the medical and surgical diseases in a single volume.

Edmund Owen's "Surgical Diseases of Children" (Cassell, 21s.), and D'Arcy Power's "Surgical Diseases of Children and their Treatment by Modern Methods" (Lewis, 10s. 6d.), are well illustrated, and give the views of experienced surgeons who have been attached for many years to large children's hospitals.

*Nervous Diseases.*—Sir Wm. Gowers' large work (Churchill, 35s.) is the standard authority on this subject. Clutterbuck's "Nerve Diseases" (Scientific Press, Ltd., 3s. net) will be found of service to students.

*Insanity.*—The manuals of Savage (Cassell, 9s.) and Clouston (Churchill, 14s.) are the most popular, and will be found sufficient for the needs of student and general practitioner.

*Diseases of the Chest.*—The works of Kingston Fowler, Harris and Beale (Lewis, 10s. 6d.), and Samuel West (Churchill, 2 vols., 36s.), can be recommended.

*Diseases of the Skin.*—Sir Malcolm Morris's small manual (Cassell, 10s. 6d.) will be found well suited to the needs of student and practitioner.

*Diseases of the Eye.*—The handbooks by Jessop (Churchill, 9s. 6d.), by Swanzy and Werner (Lewis, 12s. 6d.), and by May and Worth are perhaps to-day the most popular students' works on Ophthalmology.

*Diseases of the Nose and Throat.*—Parker's "Guide" (Arnold, 18s.) is a recent work which is thoroughly clear and practical. Mark Hovell's "Treatise on Diseases of the Ear and Naso-Pharynx" (Churchill) gives a very thorough account of aural surgery, and of affections of the nose and pharynx; while William Lamb's "Guide to the Examination of the Throat, Nose, and Ear" (Ballière, Tindall, and Cox, 5s.) is practical and reliable.

*Abdominal Surgery.*—In this large field Lockwood's "Appendicitis" (Macmillan, 10s.), and Treves' classical "Intestinal Obstruction" (Cassell, 10s. 6d.) suggest themselves as being especially valuable; while the works on Diseases of the Liver and Gall-bladder by Rollestone and by Waring are among the best in the language.

*Diseases of the Rectum.*—In this speciality the works of Harrison Cripps (Churchill, 12s. 6d.), and of Goodsall and Miles (Longmans, 7s. 6d.) deserve notice, while among the smaller books that by F. C. Wallis can be recommended.

*Orthopædic Surgery.*—"Deformities: A Treatise on Orthopædic Surgery," by A. H. Tubby (Macmillan, 17s.) is certainly the best and most complete text-book by an English surgeon in this department of surgery. It may be supplemented by "The Surgery of Paralyses," by Tubby and Jones. Mr. Jackson Clarke's "Text-book" of Orthopædics (Cassell, 21s.) can also be recommended.

*Anæsthetics.*—The large work by Hewitt (Macmillan, 15s. net) will repay careful study; but for the ordinary requirements of students and practitioners Dudley Buxton's volume (Lewis, 7s. 6d.) will be suitable; and those who desire a concise and very practical little handbook cannot do better than obtain Boyle's "Practical Anæsthetics" (Oxford Medical Manuals, 5s. net).

*Medical Electricity.*—The best book on this subject is that written by Lewis Jones (Lewis, 10s. 6d.).

THE HOSPITAL September 5, 1908.

